# Mink is a highly susceptible host species to circulating human and avian influenza viruses

**DOI:** 10.1080/22221751.2021.1899058

**Published:** 2021-03-19

**Authors:** Honglei Sun, Fangtao Li, Qingzhi Liu, Jianyong Du, Litao Liu, Haoran Sun, Chong Li, Jiyu Liu, Xin Zhang, Jizhe Yang, Yuhong Duan, Yuhai Bi, Juan Pu, Yipeng Sun, Qi Tong, Yongqiang Wang, Xiangjun Du, Yuelong Shu, Kin-Chow Chang, Jinhua Liu

**Affiliations:** a Key Laboratory of Animal Epidemiology and Zoonosis, Ministry of Agriculture, College of Veterinary Medicine, China Agricultural University, Beijing, People's Republic of China; b Department of Biostatistics, University of Michigan, Ann Arbor, Michigan, USA; c Chinese Academy of Sciences (CAS) Key Laboratory of Pathogenic Microbiology and Immunology, Center for Influenza Research and Early-Warning (CASCIRE), Institute of Microbiology, Beijing, People's Republic of China; d School of Public Health (Shenzhen), Sun Yat-sen University, Guangdong, People's Republic of China; e School of Veterinary Medicine and Science, University of Nottingham, Nottingham, UK

**Keywords:** Mink (*Mustelidae*), human influenza virus, avian influenza virus, surveillance, reassortment

## Abstract

Pandemic influenza, typically caused by the reassortment of human and avian influenza viruses, can result in severe or fatal infections in humans. Timely identification of potential pandemic viruses must be a priority in influenza virus surveillance. However, the range of host species responsible for the generation of novel pandemic influenza viruses remains unclear. In this study, we conducted serological surveys for avian and human influenza virus infections in farmed mink and determined the susceptibility of mink to prevailing avian and human virus subtypes. The results showed that farmed mink were commonly infected with human (H3N2 and H1N1/pdm) and avian (H7N9, H5N6, and H9N2) influenza A viruses. Correlational analysis indicated that transmission of human influenza viruses occurred from humans to mink, and that feed source was a probable route of avian influenza virus transmission to farmed mink. Animal experiments showed that mink were susceptible and permissive to circulating avian and human influenza viruses, and that human influenza viruses (H3N2 and H1N1/pdm), but not avian viruses, were capable of aerosol transmission among mink. These results indicate that farmed mink could be highly permissive “mixing vessels” for the reassortment of circulating human and avian influenza viruses. Therefore, to reduce the risk of emergence of novel pandemic viruses, feeding mink with raw poultry by-products should not be permitted, and epidemiological surveillance of influenza viruses in mink farms should be urgently implemented.

## Introduction

Influenza A virus epidemics occur regularly worldwide and are estimated to cause 3–5 million cases of severe illness, with 290,000–650,000 deaths each year [1]. Periodic pandemics can be much worse in terms of scale, morbidity and mortality. Historical pandemic influenza viruses, such as the 1957 H2N2, 1968 H3N2, and 2009 H1N1/pdm virus, were all derived from novel reassortant viruses [2,3]. Host species that are susceptible to both human and animal influenza viruses could, therefore, serve as “mixing vessels,” as has been described for pigs [4], through co-infections to generate novel reassortant viruses with the potential to cause pandemics. Mink, a fur-bearing animal, and the related ferret are members of the family *Mustelidae*. The ferret has been widely accepted as an excellent animal model for understanding the virulence and transmission of influenza viruses as it shares similar lung physiology and sialic acid (SA) receptor distribution to humans [5,6]. Similar to ferrets, the SA receptors SA-α-2,3-Gal and SA-α-2,6-Gal are both found in the respiratory tracts of mink [7]. Since 1984, several influenza A virus subtypes, such as avian influenza viruses (AIVs) H10N4, H5N1, and H9N2, and human influenza viruses H3N2 and H1N1/pdm [7–11], have been isolated from mink, indicating that the host species could serve as an intermediate influenza virus host in transmission between poultry and humans. However, due to a lack of systematic surveillance of influenza viruses circulating in mink, their contribution to influenza virus evolution and susceptibility to prevailing avian and human influenza viruses are unknown.

In recent years, mink farming in China has grown rapidly, with a corresponding increase in the use of animal feed, which primarily comprises raw poultry by-products. Influenza epidemiology data have indicated that there are three major subtypes of influenza viruses prevalent in poultry populations in China: H5N6, H7N9, and H9N2 [12–14]; in human populations, the predominant subtypes are H1N1/pdm and H3N2 [15]. Thus, there may be a possibility that farmed mink are regularly exposed to human and avian influenza viruses. As mink in China are not vaccinated against influenza viruses, it is relatively straightforward to perform sero-surveillance for influenza virus infections in this species. In this study, we conducted serological surveys for avian and human influenza virus infections in farmed mink and determined the susceptibility of mink to the prevailing avian and human influenza virus subtypes.

## Materials and methods

### Ethical compliance

All animal experiments were approved by the Beijing Association for Science and Technology (approval SYXK [Beijing] 2007-0023) and conducted in accordance with the Beijing Laboratory Animal Welfare and Ethics guidelines, as issued by the Beijing Administration Committee of Laboratory Animals, and in accordance with the China Agricultural University Institutional Animal Care and Use Committee guidelines (SKLAB-B-2010-003).

### Mink blood sampling and influenza virus antibody detection

Blood samples of mink were collected at slaughter, mostly from February to March and August to October according to animal slaughter plans. From September 2016 to March 2019, a total of 2455 blood samples were collected from 34 mink farms in northern China, of which 1772 were located in Shandong province (which produces more than 50% of the mink in China), and 683 were from Hebei province. For each group of 500–3000 mink, blood samples were collected on the same day from 20–150 mink (a 5% sample). This provided a 95% confidence level, that is, if the minimum prevalence of seropositive mink was 10%, at least one seropositive mink would be identified. Detailed production information was collected from each group tested which included age, feed type, location, and group size. Haemagglutination inhibition (HI) assays were performed as previously described [16]. Based on the World Health Organization (WHO) guideline for vaccine evaluation, HI antibody titres of ≥40 would be considered positive. Virus neutralization (VN) assay was used to confirm positive HI results. VN antibody titres of ≥10 were considered positive. Mink sera were considered seropositive only if they were both HI- and VN-positive (HI+/VN+). From 2016 to 2019, H1N1/pdm and H3N2 influenza viruses were prevalent in human populations in northern China [15]. The AIV subtypes H5N6, H7N9, and H9N2 are endemic in domestic poultry [12,13]. Thus, human H1N1/pdm and H3N2 viruses, and avian H5N6, H7N9, and H9N2 viruses were chosen as diagnostic antigens for the HI and VN assays. Taking into consideration antigenic drift of influenza viruses over time, A/Hebei/1104/2016 (H1N1/pdm), A/Beijing/1230/2016 (H3N2), A/duck/Eastern China/S0711/2014 (H5N6), A/chicken/Henan/KF/2014 (H7N9), and A/chicken/Shandong/1/2016 (H9N2) viruses were used to detect seroconversion in samples from 2016 to 2017; A/Beijing/0212/2018 (H1N1/pdm), A/Tianjin/0122/2018 (H3N2), A/chicken/Northern China/F0130c/2018 (H5N6), A/chicken/Hebei/0417/2018 (H7N9), and A/chicken/Shandong/0322/2018 (H9N2) were used on sera from 2018 to 2019.

### Mink influenza virus infection and transmission

Four-month-old female mink, weighing 1000–1200 g, were purchased from a farm in Yantai, Shandong province. All were seronegative for currently circulating influenza viruses (H1, H3, H5, H7, and H9). Three mink in each group were anaesthetized with ketamine (20 mg/kg) and xylazine (1 mg/kg), and inoculated intranasally with 10^6^ 50% tissue culture infective dose (TCID_50_) of test virus in a 1 mL volume (500 μL per nostril). The animals were subsequently euthanised at 4 days post-infection (dpi), and nasal turbinate, trachea, lung, liver, kidney, spleen, intestine, and brain samples were collected for virus titration in Madin–Darby canine kidney (MDCK) cells. Nasal turbinate, trachea, and lung tissues were also used for histopathological analysis and immuno-detection of viral nucleoprotein. In virus transmission, groups of three mink were anaesthetized and inoculated intranasally with 10^6^ TCID _50_ of virus. The following day, a respiratory-droplet contact mink was housed in a wire frame cage 5 cm from the infected donor mink. To monitor virus shedding, nasal washes were collected from all animals every other day for 12 days and titrated for virus in MDCK cells. Sera were collected from both the inoculated and contact animals at 14 dpi. Seroconversion was analysed using HI assays. Clinical signs, temperature and body weight were recorded daily for all animals. A/Beijing/0212/2018 (H1N1/pdm), A/Tianjin/0122/2018 (H3N2), A/mink/Northern China/F0130 m/2018 (H5N6, a highly pathogenic AIV [HPAIV]), A/chicken/Hebei/0417/2018 (H7N9, a low pathogenicity avian influenza virus [LPAIV]), and A/chicken/Shandong/0322/2018 (H9N2) viruses were used for mink infection. All experiments with H5 and H7 subtype viruses were performed in biosafety level 3 containments.

### Statistical analyses

The relationship between seroprevalence of human influenza viruses in mink and human infection cases was evaluated using Spearman rank correlation coefficient (*rho*, *ρ*). The degree of correlation was considered weak if *ρ* was <0.4, moderate if *ρ* was ≥0.4 and <0.6, and strong if *ρ* was ≥0.6. Human case data were obtained from the weekly influenza surveillance reports of the Chinese Centre for Disease Control and Prevention (CDC) from 2016 to 2019 [15]. Pearson’s chi-squared test or Fisher's exact test was used to assess significant differences in seroprevalence between the groups with different feed types. For all analyses, a *P-*value of <.05 was considered to be statistically significant. All analyses were performed using SPSS software version 19.0 (IBM Corporation, Armonk, NY, USA).

## Results

### Seroprevalence of antibodies against influenza viruses in farmed mink

**Figure 1.**
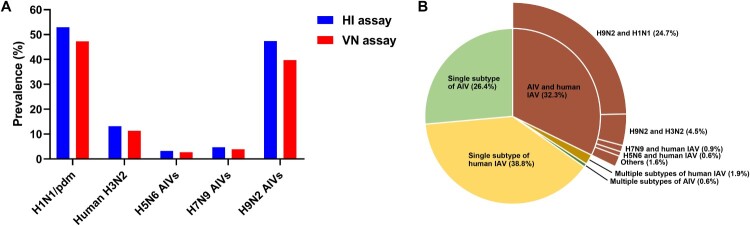
Seroprevalence of influenza viruses in farmed mink in northern China. (A) Seroprevalence of influenza viruses in mink by combined HI and VN assays. A total of 2455 serum samples were assayed by HI (titres of ≥40 were considered positive). All HI positive samples were confirmed by VN assays (titres of ≥10 were considered positive). (B) Breakdown of positive samples by virus subtypes. Details of the number of positive samples are shown in Table S1. Note: HI: haemagglutination inhibition; VN: virus neutralization.

**Figure 2.**
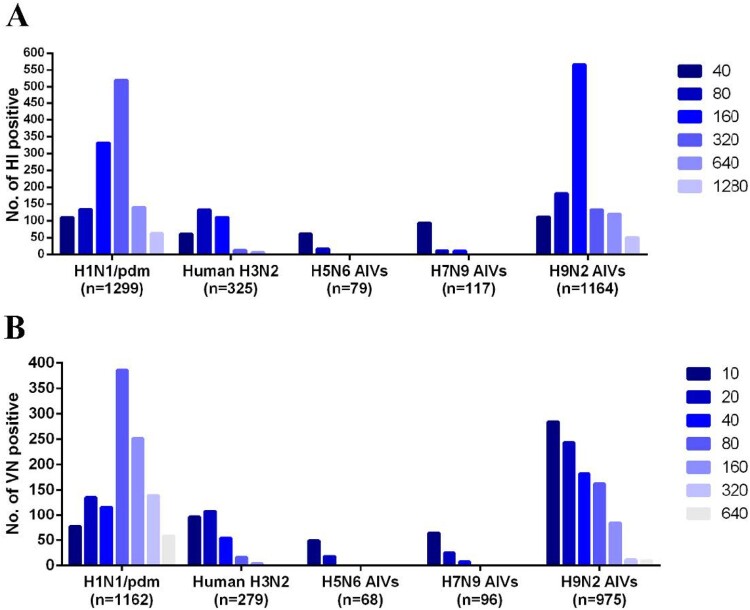
Antibody titres in serum samples from farmed mink that were positive for influenza viruses. (A) HI titres in samples positive for human H1N1/pdm and H3N2 viruses, or avian H5N6, H7N9 and H9N2 viruses from a total of 2455 samples (titres of ≥40 were considered positive). (B) VN titres in samples positive for human H1N1/pdm and H3N2 viruses, or avian H5N6, H7N9, or H9N2 viruses from a total of 2108 HI-positive samples (titres of ≥10 were considered positive). The number of positive serum samples for each virus is indicated.

The overall seroprevalence of human and avian influenza viruses in the collected mink samples was 76.8% (1,885/2,455 [positive for both HI and VN assays, HI+/VN+]), of which 47.3% (1,162/2,455) and 11.4% (279/2,455) were positive for H1N1/pdm and H3N2 influenza viruses, respectively; and 2.8% (68/2,455), 3.9% (96/2,455), and 39.7% (975/2,455) were positive for avian viruses H5N6, H7N9, and H9N2, respectively (Figure 1(A) and Figure 2).

Further analysis of co-infection prevalence in the 1885 HI+/VN+ samples showed that 34.7% (655/1885) of the samples were seropositive for multiple influenza virus subtypes, of which 32.3% (608/1885) contained both avian and human influenza antibodies (Figure 1(B) and Supplementary Figure 1). Specifically, the seroprevalence in mink for avian H9N2 and human H1N1/pdm viruses was 26.2% (494/1885), seroprevalence for avian H5N6 and human influenza viruses was 0.8% (15/1885), and seroprevalence for avian H7N9 and human influenza viruses was 1.2% (23/1885) (Supplementary Table 1).

### Association between incidence of human influenza virus infection in humans and corresponding seroprevalence in farmed mink

**Figure 3.**
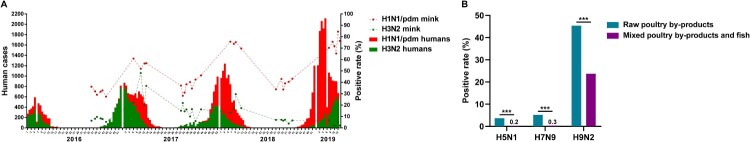
Correlational analysis of mink infection with human and avian influenza viruses. (A) Statistical analysis of the number of human seasonal influenza A (H1N1/pdm and H3N2) cases and the seroprevalence of influenza virus infection in farmed mink in northern China from 2016 to 2019. Coloured columns indicate weekly number of human cases of influenza virus infection from the influenza surveillance reports of China CDC from 2016 to 2019. Dashed lines show the seroprevalence of H1N1/pdm and H3N2 viruses in different mink farms. (B) Comparison of the seroprevalence values according to the types of food used to feed mink in farms. ***P < .001, between farms feeding mink with poultry by-products only and those feeding mink with both poultry by-products and fish.

Analysis of monthly seroprevalence data for all viruses found that the seroprevalence of human influenza virus in mink correlated with circulating virus levels in humans; it was high from February to March and relatively low from August to October (Supplementary Table 2). We further analysed the correlations of the incidence of human influenza virus infections between mink and human populations. Cumulative human cases of H1N1/pdm virus infections had increased annually from 2016 (Figure 3(A)). Seroprevalence of H1N1/pdm virus in mink followed the same rising trend, peaking at 72.0% in 2019 (Figure 3(A)), and was positively correlated with the incidence of human infection (*P* = .005, *ρ* = .746) (Table 1). Human cases of human H3N2 virus infections peaked in 2017 followed by subsequent decline. Similarly, the seroprevalence of H3N2 virus in mink was at its highest in 2017 (at 20.2%), which was followed by decline. There was no significant correlation between the seroprevalence of H3N2 virus antibodies in mink and incidence of H3N2 virus infections in humans (Table 1). However, given that influenza seroconversion in mink would lag behind those in human populations, and if this lag period of one or two months is taken into account, the correlation between the two host populations for H3N2 virus infection was statistically significant (Table 1).

**Table 1.** Correlations of seroprevalence of human influenza viruses between mink and human populations.

Number of months lagged	H1N1/pdm		H3N2	
	*rho*^a^	*P* value^a^	*rho*	*P* value
0	0.746	.*005*	0.445	.147
1	0.739	.*006*	0.616	.*033*
2	0.789	.*002*	0.674	.*016*

Note: Statistically significant differences (*P* < .05) in italics.

^a^ Spearman correlation test.

### Association between feed type and avian influenza virus infection in farmed mink

Previous research found that influenza virus could be transmitted from swine to mink following feeding animals with a ration composed of uncooked meat by-products of swine [11]. Here, we examined whether feed was a possible route for AIV transmission to farmed mink. Mink are usually fed with raw poultry by-products (offal and carcasses of poultry) from two months old. For better reproduction performance, some mink farms use fish products supplemented with 20–30% poultry by-products. The majority of farms investigated (24/34) used only poultry by-products, and the remaining 10 farms used both poultry by-products and fish (mixed-feed farms) (Supplementary Table 3). In farms that used only poultry by-products, the seroprevalence values of avian H5N6, H7N9, and H9N2 viruses were 3.7%, 5.2%, and 45.4%, respectively. These were significantly higher (*P *< .001) than the seroprevalence values in the mixed-feed farms, which were 0.2%, 0.3%, and 23.7% for H5N6, H7N9, and H9N2 viruses, respectively (Figure 3(B)).

### Susceptibility of mink to circulating avian and human influenza viruses

**Figure 4.**
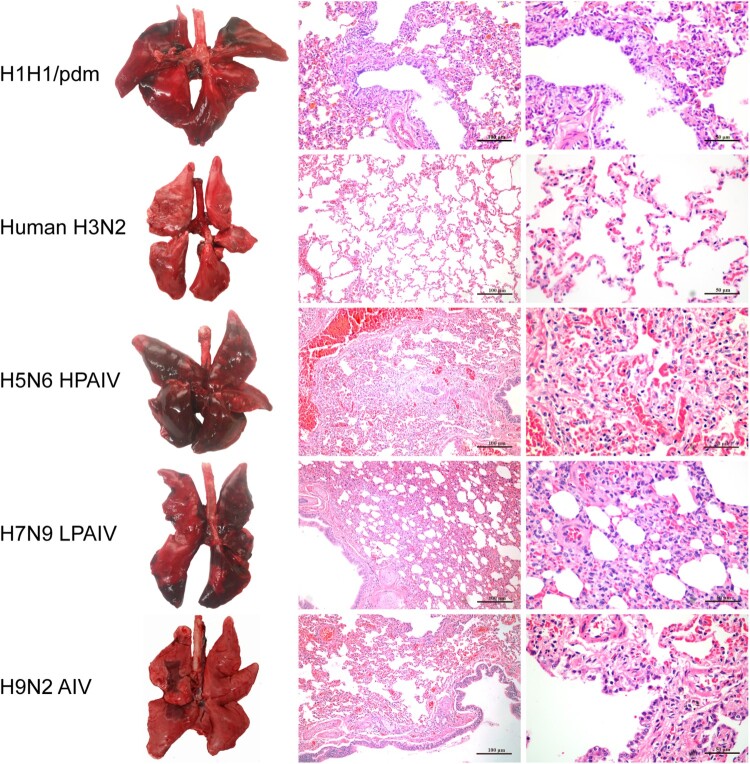
Gross pathology and histopathology of the lungs of mink infected with human (H1N1/pdm and H3N2) and avian (H5N6, H7N9, and H9N2) influenza viruses at 4 dpi. Lungs of mink with H1N1/pdm, H9N2, and H7N9 influenza virus infections showed mild to moderate pulmonary consolidation with varying degrees of bronchitis. Few remarkable pathological findings were obtained from the lungs of mink infected with the H3N2 virus. In contrast, the H5N6 influenza virus caused severe lesions with extensive pulmonary consolidation, peribronchiolitis and bronchopneumonia characterised by oedema and infiltration of inflammatory cells. Images shown are representative of three mink from three independent experiments.

**Figure 5.**
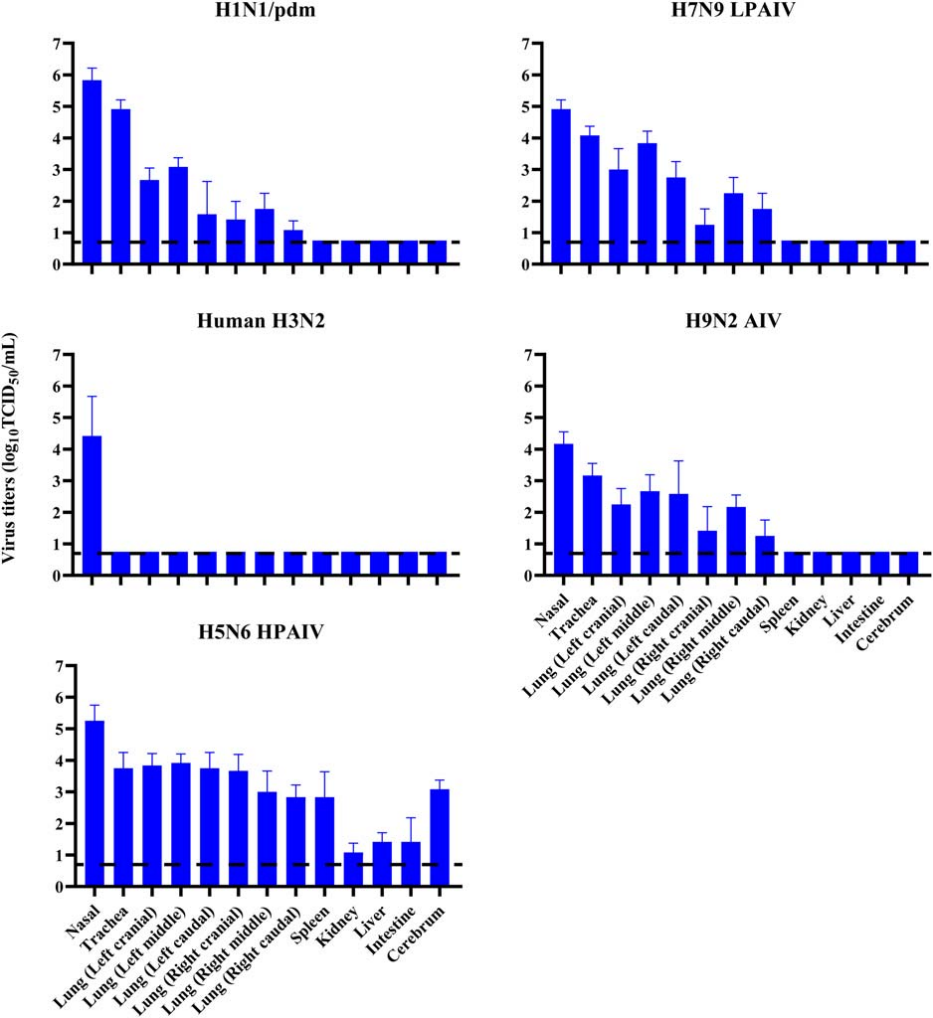
Virus replication in mink infected with avian and human influenza viruses. Mink were inoculated intranasally with 10^6^ TCID_50_ of indicated viruses. Three mink per group were euthanised at 4 dpi, and samples of nasal turbinate, trachea, lung, spleen, liver, kidney, intestine and cerebrum were collected from each mink for virus titration by TCID_50_ assays on MDCK cells. Systemic spread of H5N6 virus was detected in all organs examined. All values shown are means ± SD from three independent experiments for each sample. Dashed lines indicate the lower limits of detection.

**Figure 6.**
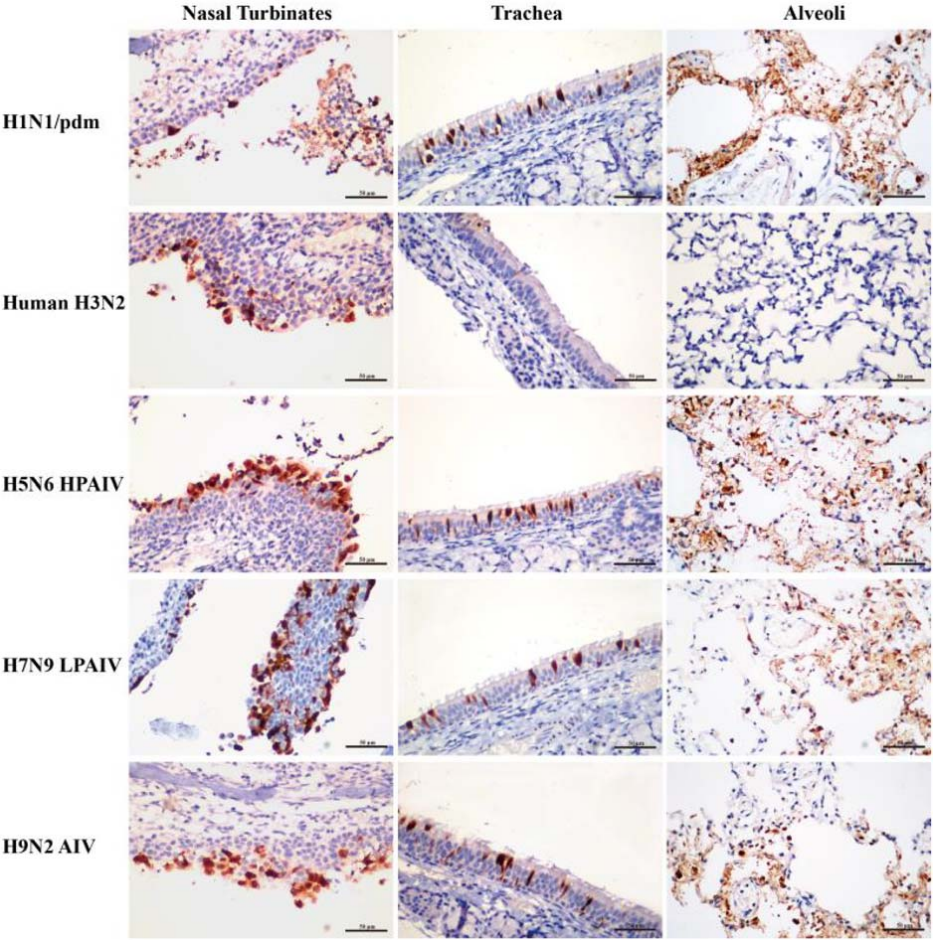
Detection of viral nucleoprotein in the respiratory tract of mink infected with influenza viruses at 4 dpi. Tissue sections of nasal turbinate, trachea and lung (alveoli) were immunostained for viral nucleoprotein (red-brown staining). Extensive distribution of H1N1/pdm, H5N6, H7N9, and H9N2 influenza virus were evident in the nasal turbinate, trachea, and lung. In contrast, the H3N2 virus was mainly restricted to the nasal turbinate.

To determine host susceptibility, mink were intranasally infected with human (H1N1/pdm and H3N2) and avian (H5N6, H7N9, and H9N2) influenza viruses. Clinically, mink infected with human H3N2 virus were asymptomatic, while those infected with H1N1/pdm, H7N9, and H9N2 influenza viruses exhibited transient signs of pyrexia (Supplementary Figure 2(A)), sneezing and nasal discharge. H5N6 virus was the most pathogenic, and infected mink displayed severe pyrexia and weight loss (>15%), which led to the euthanasia of all three mink at 10 dpi (Supplementary Figure 2(B)). No apparent abnormality was found in the lungs of mink with H3N2 virus infection; however, multifocal areas of pulmonary consolidation were present in the lungs of mink infected with H1N1/pdm, H7N9, and H9N2 viruses (Figure 4). Consistent with the clinical severity, H5N6 virus infections caused the most severe gross and histopathological changes in the lungs (Figure 4). Virus shedding up to 6 dpi was evident from infected mink for all avian and human influenza viruses, with a virus titre range of 10^3.5^–10^5.5^ TCID_50_/mL based on nasal washes. With avian H5N6 and H9N2 viruses, shedding continued to be detected at 8 dpi (Supplementary Table 4). Virus spread to other tissues was consistent with the severity of infection. Human H3N2 virus was detected only in the nasal turbinate, whereas H1N1/pdm, H7N9, and H9N2 viruses were detected throughout the respiratory tract (Figures 5 and 6). The systemic spread of the H5N6 virus was evident by its presence in all organs examined (Figures 5 and 6).

### Transmission of human influenza viruses among mink

**Figure 7.**
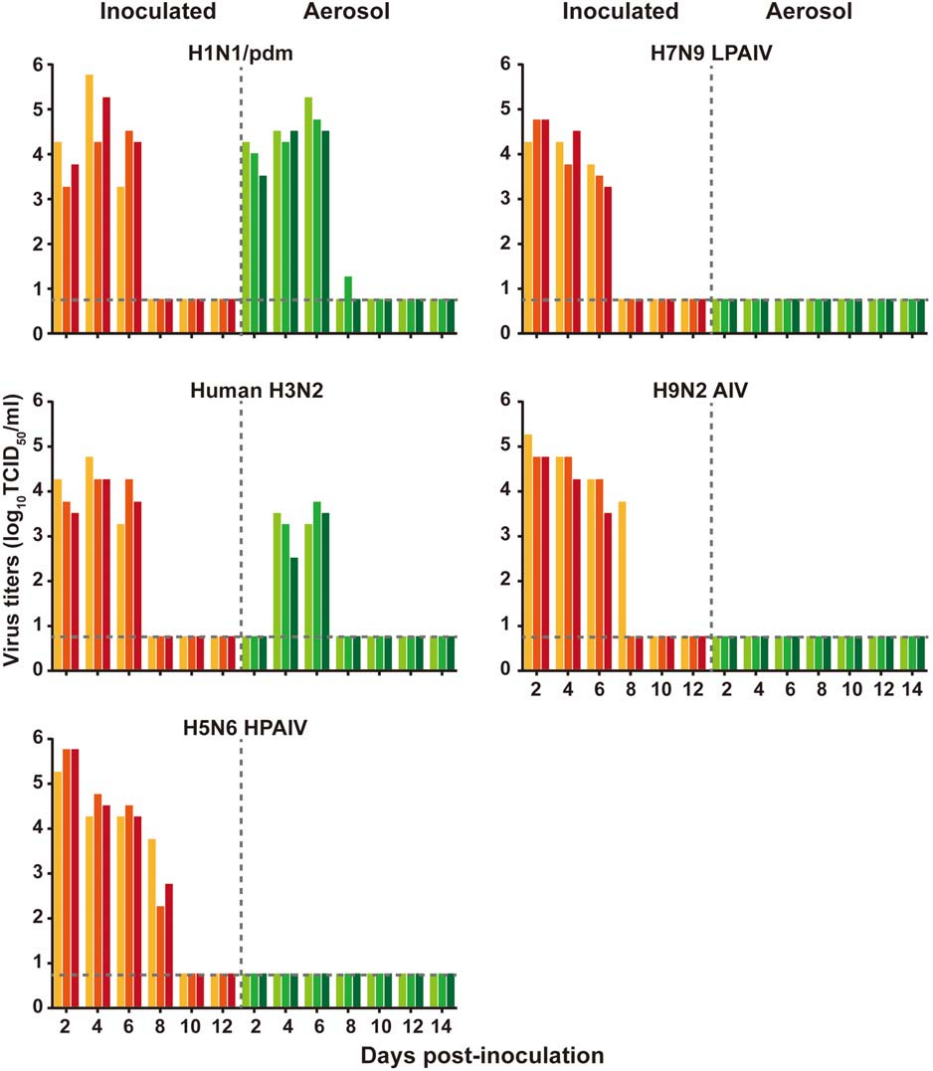
Aerosol transmission of human influenza viruses between mink. Groups of three mink were inoculated intranasally with 10^6^ TCID_50_ of the viruses indicated. The following day, a respiratory-droplet contact mink was housed in a wire frame cage 5 cm from infected donor mink cage. Nasal washes for virus shedding detection were collected every two days from all animals. Virus titres were determined by TCID_50_ assays on MDCK cells. Each coloured bar represents the virus titre in a single animal. Horizontal dashed lines indicate the lower limits of virus detection.

To assess airborne transmissibility of human and avian influenza A viruses among mink, naïve mink were placed 5 cm away, in groups of three in separate wire cages, from infected mink 24 h post-infection. Human H1N1/pdm and H3N2 viruses were readily transmitted from mink that were infected earlier, via aerosol, to naïve mink at 2 and 4 dpi, respectively, with subsequent seroconversion (Figure 7 and Supplementary Table 5). No airborne transmission to naïve mink was detected with avian H5N6, H7N9, and H9N2 viruses (Figure 7 and Supplementary Table 5).

## Discussion

We found that farmed mink in China were commonly infected with avian and human influenza viruses, of which human H1N1/pdm and avian H9N2 influenza virus infections were the most prevalent. The high seroprevalence of combined avian and human influenza viruses suggests a strong likelihood of co-infections and thus farmed mink could serve as “mixing vessels,” as in pigs, for the generation of novel reassortant viruses. Our host susceptibility analysis found that mink are susceptible and permissive to circulating avian (H7N9, H5N6, and H9N2) and human (H3N2 and H1N1/pdm) influenza A viruses. Only the human influenza viruses, not avian viruses, were capable of aerosol transmission among mink, suggesting that mink are similar to ferrets as conducive hosts for human influenza virus replication. We previously found that the avian H9N2 virus subtype shows good gene compatibility in reassortment with the human H1N1/pdm virus, which in co-infection could generate novel reassortant viruses with high human infectivity [17]. Although the co-infection rates of avian H5 or H7 viruses with human H1N1/pdm or H3N2 viruses appear low, the public health threat from such reassortant viruses should not be ignored as their pandemic potential has been experimentally demonstrated [18–21].

Infection prevalence of human H1N1/pdm or H3N2 viruses in both human and mink populations strongly indicates human-to-mink virus transmission. Previous studies suggested that mammalian and avian influenza viruses could be effectively transmitted to mink through uncooked poultry or pork by-products [7,10,11,22–24]. Our correlational analysis supports the notion that feeding mink with raw poultry by-products is a major source of AIV transmission. Avian H9N2 influenza virus is the most dominant subtype in poultry [13,25] and was also the most common seropositive subtype found in farmed mink. Notably, since the introduction of the avian H7N9 subtype vaccine to chicken flocks in China in 2017 [26], no H7N9 seropositive mink was detected in 2019.

Recently, the rapid spread of the severe acute respiratory syndrome coronavirus 2 (SARS-CoV-2) infection was reported in mink farms in several countries in Europe and the Americas; millions of mink were culled to control the spread of the virus [27–29]. As mink are inherently susceptible to both influenza viruses and SARS-CoV-2, it is important to prevent mink from being infected with such viruses. Clear intervention steps that should be taken are to stop the use of raw poultry products in the prevention of influenza virus transmission to mink and to isolate all workers with respiratory symptoms from mink farms. In summary, mink as a host species is highly susceptible and permissive to circulating human and avian influenza viruses. To avoid serious public health threats from the emergence of highly pathogenic virus reassortants, basic preventative husbandry steps, such as a ban on the use of raw poultry feed, along with regular virus surveillance of mink farms should be urgently implemented.

## Supplementary Material

Supplemental_Material-clean_2021.2.28.docx
