# Supplementary material for: Mink is a highly susceptible host species to circulating human and avian influenza viruses
Source: Emerg Microbes Infect. 2021 Mar 19;10(1):472–80. doi: 10.1080/22221751.2021.1899058 (PMC7993395; doi:10.1080/22221751.2021.1899058)
Supplement: Supplemental_Material-clean_2021.2.28.docx [file TEMI_A_1899058_SM8630.docx]

**Supplemental material**

**Supplementary Table 1** Distribution of serologically positive samples according to different combinations of influenza virus subtypes.

| Characteristic | No.  positive | Prevalence  (%) |
| --- | --- | --- |
| Infected with 4 subtypes of influenza viruses |  |  |
| H1N1, H3N2, H5N6 and H9N2 | 1 | 0.05 |
| H1N1, H3N2, H7N9 and H9N2 | 3 | 0.16 |
| Total | 4 | 0.21 |
| Infected with 3 subtypes of influenza viruses |  |  |
| H1N1, H3N2 and H5N6 | 2 | 0.11 |
| H1N1, H3N2 and H7N9 | 4 | 0.21 |
| H1N1, H3N2 and H9N2 | 21 | 1.11 |
| H1N1, H5N6 and H9N2 | 1 | 0.05 |
| H1N1, H7N9 and H9N2 | 3 | 0.16 |
| H3N2, H5N6 and H9N2 | 1 | 0.05 |
| Total | 32 | 1.70 |
| Infected with 2 subtypes of influenza viruses |  |  |
| H1N1 and H3N2 | 35 | 1.86 |
| H1N1 and H5N6 | 5 | 0.27 |
| H1N1 and H7N9 | 8 | 0.42 |
| H1N1 and H9N2 | 465 | 24.67 |
| H3N2 and H5N6 | 5 | 0.27 |
| H3N2 and H7N9 | 5 | 0.27 |
| H3N2 and H9N2 | 84 | 4.46 |
| H5N6 and H9N2 | 7 | 0.37 |
| H7N9 and H9N2 | 5 | 0.27 |
| Total | 619 | 32.84 |
| Infected with single subtype of influenza virus |  |  |
| H1N1 | 614 | 32.57 |
| H3N2 | 118 | 6.26 |
| H5N6 | 46 | 2.44 |
| H7N9 | 68 | 3.61 |
| H9N2 | 384 | 20.37 |
| Total | 1230 | 65.25 |
| Infected with both human and avian influenza viruses | 608 | 32.25 |
| Infected with multiple subtypes of human influenza viruses | 35 | 1.86 |
| Infected with multiple subtypes of avian influenza viruses | 12 | 0.64 |
| Infected with single subtype of human influenza virus | 732 | 38.83 |
| Infected with single subtype of avian influenza virus | 498 | 26.42 |
| Total number of positive samples | 1885 | 100 |

**Supplementary Table 2** Monthly seroprevalence of human and avian influenza viruses in mink farms

| Collection  Month | No.  farms | No.  samples | No. positive (%) | | | | |
| --- | --- | --- | --- | --- | --- | --- | --- |
|  |  |  | H1N1/pdm | Human H3N2 | H9N2 AIVs | H5N6 AIVs | H7N9 AIVs |
| 2 | 1 | 33 | 19 (57.6) | 12 (36.4) | 8 (24.2) | 0 (0) | 0 (0) |
| 3 | 13 | 888 | 608 (68.5) | 102 (11.5) | 294 (33.1) | 14 (1.6) | 13 (1.5) |
| 8 | 7 | 451 | 149 (33.0) | 65 (14.4) | 187 (41.5) | 23 (5.1) | 37 (8.2) |
| 9 | 8 | 679 | 244 (35.9) | 66 (9.7) | 301 (44.3) | 24 (3.5) | 34 (5.0) |
| 10 | 5 | 404 | 142 (35.1) | 34 (8.4) | 185 (45.8) | 7 (1.7) | 12 (3.0) |

**Supplementary Table 3** Summary of viral serology and main feed source in mink farms

| Collection |  | No. |  | No. positive (% positive) | | | | |
| --- | --- | --- | --- | --- | --- | --- | --- | --- |
| date | Location | samples | Feed type | H1N1/pdm | Human H3N2 | H9N2 AIVs | H5N6 AIVs | H7N9 AIVs |
| 2016.9 | Shandong | 63 | Poultry by-products | 21 (33.3) | 4 (6.3) | 34 (54.0) | 0 (0) | 0 (0) |
| 2016.9 | Shandong | 50 | Poultry by-products | 15 (30.0) | 4 (8.0) | 23 (46.0) | 0 (0) | 0 (0) |
| 2016.9 | Shandong | 62 | Poultry by-products | 18 (29.0) | 5 (8.1) | 28 (45.2) | 6 (9.7) | 23 (37.1) |
| 2016.10 | Shandong | 60 | Poultry by-products | 18 (30.0) | 5 (8.3) | 31 (51.7) | 0 (0) | 0 (0) |
| 2016.10 | Hebei | 64 | Poultry by-products | 21 (32.8) | 5 (7.8) | 30 (46.9) | 2 (3.1) | 7 (10.9) |
| 2016.10 | Hebei | 113 | Poultry by-products | 29 (25.7) | 6 (5.3) | 58 (51.3) | 2 (1.8) | 4 (3.5) |
| 2017.2 | Shandong | 33 | Mixed poultry by-products and fish | 19 (57.6) | 12 (36.4) | 8 (24.2) | 0 (0) | 0 (0) |
| 2017.3 | Shandong | 27 | Mixed poultry by-products and fish | 14 (51.9) | 13 (48.1) | 6 (22.2) | 0 (0) | 0 (0) |
| 2017.3 | Shandong | 55 | Poultry by-products | 30 (54.5) | 18 (32.7) | 28 (50.9) | 4 (7.3) | 7 (12.7) |
| 2017.3 | Shandong | 90 | Poultry by-products | 49 (54.4) | 22 (24.4) | 31 (34.4) | 3 (3.3) | 4 (4.4) |
| 2017.8 | Shandong | 62 | Mixed poultry by-products and fish | 22 (35.5) | 20 (32.3) | 16 (25.8) | 0 (0) | 2 (3.2) |
| 2017.8 | Shandong | 64 | Poultry by-products | 17 (26.6) | 14 (21.9) | 28 (43.8) | 7 (10.9) | 5 (7.8) |
| 2017.8 | Hebei | 55 | Poultry by-products | 17 (30.9) | 8 (14.5) | 26 (47.2) | 4 (7.3) | 21 (38.2) |
| 2017.8 | Hebei | 62 | Poultry by-products | 21 (33.9) | 9 (14.6) | 28 (45.2) | 3 (4.8) | 7 (11.3) |
| 2017.9 | Shandong | 142 | Poultry by-products | 55 (38.7) | 16 (11.3) | 66 (46.5) | 8 (5.6) | 5 (3.5) |
| 2017.9 | Shandong | 110 | Poultry by-products | 37 (33.6) | 18 (16.4) | 46 (41.8) | 5 (4.5) | 4 (3.6) |
| 2017.9 | Shandong | 78 | Poultry by-products | 33 (42.3) | 10 (12.8) | 37 (47.4) | 0 (0) | 2 (2.6) |
| 2017.10 | Shandong | 74 | Poultry by-products | 34 (45.9) | 12 (16.2) | 28 (37.8) | 0 (0) | 0 (0) |
| 2018.3 | Shandong | 61 | Mixed poultry by-products and fish | 44 (72.1) | 8 (13.1) | 14 (23.0) | 0 (0) | 0 (0) |
| 2018.3 | Shandong | 53 | Mixed poultry by-products and fish | 38 (71.7) | 9 (17.0) | 12 (22.6) | 0 (0) | 0 (0) |
| 2018.3 | Hebei | 44 | Poultry by-products | 32 (72.7) | 13 (29.5) | 27 (61.4) | 3 (6.8) | 2 (4.5) |
| 2018.3 | Hebei | 23 | Poultry by-products | 16 (69.6) | 4 (17.4) | 14 (60.9) | 0 (0) | 0 (0) |
| 2018.8 | Shandong | 71 | Poultry by-products | 23 (32.4) | 5 (7.0) | 35 (49.3) | 4 (5.6) | 0 (0) |
| 2018.8 | Shandong | 42 | Poultry by-products | 18 (42.9) | 3 (7.1) | 16 (38.1) | 1 (2.4) | 0 (0) |
| 2018.8 | Shandong | 95 | Poultry by-products | 31 (32.6) | 6 (6.3) | 38 (40.0) | 4 (4.2) | 2 (2.1) |
| 2018.9 | Shandong | 70 | Poultry by-products | 26 (37.1) | 5 (7.1) | 26 (37.1) | 2 (2.9) | 0 (0) |
| 2018.9 | Hebei | 104 | Poultry by-products | 39 (37.5) | 4 (3.8) | 41 (39.4) | 3 (2.9) | 0 (0) |
| 2018.10 | Hebei | 93 | Poultry by-products | 40 (43.0) | 6 (6.5) | 38 (40.9) | 3 (3.2) | 1 (1.1) |
| 2019.3 | Shandong | 107 | Mixed poultry by-products and fish | 73 (68.2) | 7 (6.5) | 25 (23.4) | 0 (0) | 0 (0) |
| 2019.3 | Shandong | 73 | Mixed poultry by-products and fish | 54 (74.0) | 0 (0) | 17 (23.3) | 0 (0) | 0 (0) |
| 2019.3 | Shandong | 144 | Mixed poultry by-products and fish | 102 (70.8) | 3 (2.1) | 36 (25.0) | 1 (0.7) | 0 (0) |
| 2019.3 | Shandong | 86 | Mixed poultry by-products and fish | 55 (64.0) | 0 (0) | 19 (22.1) | 0 | 0 (0) |
| 2019.3 | Hebei | 76 | Poultry by-products | 63 (82.9) | 4 (5.3) | 39 (51.3) | 3 (3.9) | 0 (0) |
| 2019.3 | Hebei | 49 | Poultry by-products | 38 (77.6) | 1 (2.0) | 26 (53.1) | 0 (0) | 0 (0) |

**Supplementary Table 4** Virus shedding of influenza virus in mink

| Virus | Days post-infection (Nasal wash titers) | | | | | | |
| --- | --- | --- | --- | --- | --- | --- | --- |
|  | 2 | 4 | 6 | 8 | 10 | 12 | 14 |
| H1N1/pdm | 4.8±0.6^a^ | 4.7±0.2 | 4.2±0.4 | - | - | - | - |
| Human H3N2 | 4.5±0.3 | 4.2±0.4 | 3.5±0.3 | - | - | - | - |
| H5N6 HPAIV | 4.8±0.4 | 4.6±0.2 | 4.3±0.5 | 3.4±0.3 | / | / | / |
| H7N9 LPAIV | 4.3±0.3 | 3.8±0.4 | 4.7±0.4 | - | - | - | - |
| H9N2 AIV | 5.5±0.2 | 4.7±0.3 | 4.5±0.6 | 3.2±0.8 | - | - | - |

“-” indicates negative. “/” indicates not tested as mink was euthanized.

^a^Mean titer log_10_ (TCID_50_/mL) of viruses, error bars are SD.

**Supplementary Table 5** Seroconversion of influenza virus in mink

|  | Seroconversion: No. positive/No. total (HI titers)*^a^* | |
| --- | --- | --- |
| Virus | Inoculated | Respiratory contact |
| H1N1/pdm | 3/3 (160, 80, 80) | 3/3 (80, 80, 40) |
| Human H3N2 | 3/3 (80, 80, 80) | 3/3 (40, 80, 40) |
| H5N6 HPAIV | - | 0/3 |
| H7N9 LPAIV | 3/3 (80, 80, 40) | 0/3 |
| H9N2 AIV | 3/3 (160, 80, 80) | 0/3 |

*^a^*Sera were collected from mink at 14 dpi.


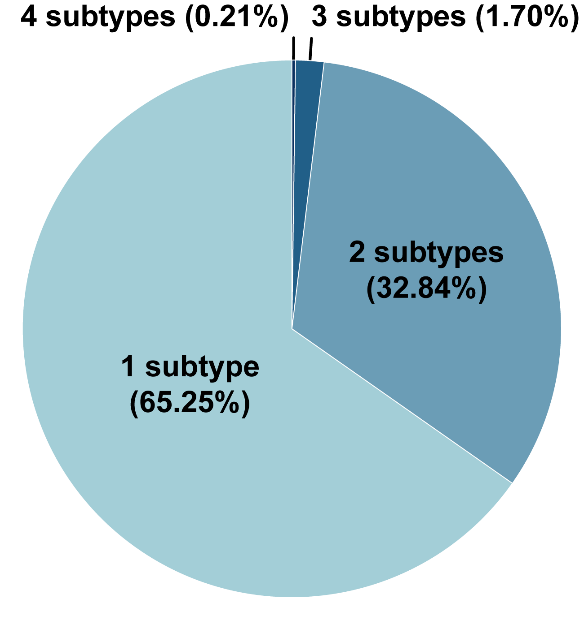


**Supplementary Figure 1.** Distribution of serologically positive samples according to the presence of different combinations of influenza virus subtypes. Details are shown in Table S1.


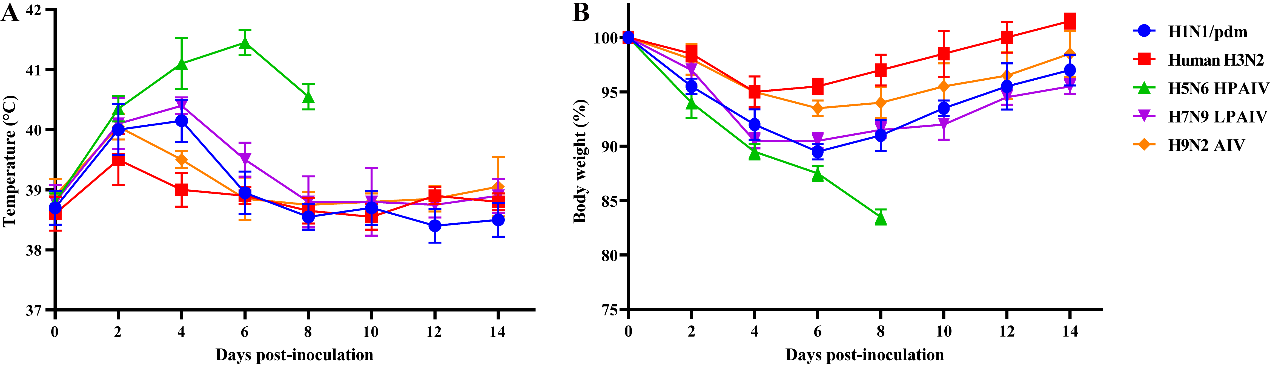


**Supplementary Figure 2.** Body temperature (A) and body weight (B) of mink following intranasal inoculation with human and avian influenza viruses. Five representative influenza viruses were selected to infect mink: A/Beijing/0212/2018 (H1N1/pdm), A/Tianjin/0122/2018 (H3N2), A/mink/Northern China/F0130m/2018 (H5N6 HPAIV), A/chicken/Hebei/0417/2018 (H7N9 LPAIV), and A/chicken/Shandong/0322/2018 (H9N2). Four-month-old female mink were intranasally inoculated with 10^6^ TCID_50_ of each test virus. Rectal temperature and body weight of infected mink were taken every 2 days. Average temperature of each group is shown with standard deviations (SD). Average group body weight with SD is presented as percentage of starting weight at day of inoculation (day 0).
